# 4-Bromo-1-[2,6-dichloro-4-(trifluoro­meth­yl)phen­yl]-5-(4-nitro­benzyl­idene­amino)-1*H*-pyrazole-3-carbonitrile

**DOI:** 10.1107/S160053680800545X

**Published:** 2008-02-29

**Authors:** Zhiping Yang, Shuyan Li

**Affiliations:** a Department of Food and Biotechnology, Zhangzhou Vocational Technical College, 363000 Zhangzhou, People’s Republic of China

## Abstract

The title compound, C_18_H_7_BrCl_2_F_3_N_5_O_2_, is an L-shaped tricyclic imine. The pyrazole ring is essentially coplanar with the nitro-substituted benzene ring [dihedral angle = 3.6 (2)°] and approximately perpendicular to the trifluoro­methyl­substituted ring [dihedral angle = 88.5 (2)°].

## Related literature

For related literature, see: Philippe (1997 ▶, 2000 ▶); Zhong *et al.* (2005 ▶).
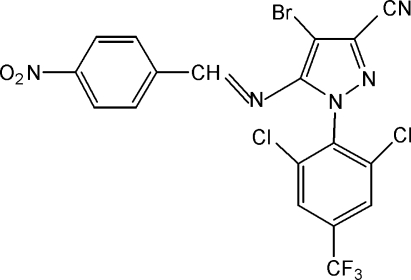


## Experimental

### 

#### Crystal data

C_18_H_7_BrCl_2_F_3_N_5_O_2_
*M* *_r_* = 533.10
Monoclinic, 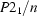

*a* = 8.0925 (7) Å
*b* = 13.0406 (10) Å
*c* = 19.7165 (16) Å
β = 100.753 (2)°
*V* = 2044.2 (3) Å^3^
*Z* = 4
Mo *K*α radiation
μ = 2.33 mm^−1^
*T* = 298 (2) K
0.27 × 0.24 × 0.22 mm

#### Data collection

Bruker APEX area-detector diffractometer
Absorption correction: multi-scan (*SADABS*; Bruker, 2002 ▶) *T*_min_ = 0.572, *T*_max_ = 0.629
12155 measured reflections
4437 independent reflections
3331 reflections with *I* > 2σ(*I*)
*R*_int_ = 0.024

#### Refinement

*R*[*F* ^2^ > 2σ(*F* ^2^)] = 0.041
*wR*(*F* ^2^) = 0.110
*S* = 1.07
4437 reflections
280 parameters
H-atom parameters constrained
Δρ_max_ = 0.56 e Å^−3^
Δρ_min_ = −0.38 e Å^−3^

### 

Data collection: *SMART* (Bruker, 2002 ▶); cell refinement: *SAINT* (Bruker, 2002 ▶); data reduction: *SAINT*; program(s) used to solve structure: *SHELXS97* (Sheldrick, 2008 ▶); program(s) used to refine structure: *SHELXL97* (Sheldrick, 2008 ▶); molecular graphics: *SHELXTL* (Sheldrick, 2008 ▶); software used to prepare material for publication: *SHELXL97*.

## Supplementary Material

Crystal structure: contains datablocks global, I. DOI: 10.1107/S160053680800545X/fl2181sup1.cif

Structure factors: contains datablocks I. DOI: 10.1107/S160053680800545X/fl2181Isup2.hkl

Additional supplementary materials:  crystallographic information; 3D view; checkCIF report

## Figures and Tables

**Fig. 1.**
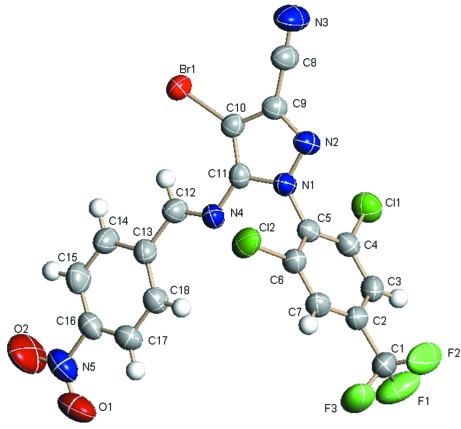
The molecular structure of (I) showing the atom numbering scheme and displacement ellipsoids at 50% probability level.

## supplementary crystallographic information

### Comment

The title compound, (I) (Fig. 1), is similar to the effective insecticides used to treat animals such as cows and sheep (Philippe, 1997, 2000). Earlier we reported on a structure with the same ring system but without the Br substituent on the pyrazole ring (Zhong *et al.*, 2005). That molecule had an overall U shape and exhibited some π-π interactions between the pyrazole and nitro benzene rings. (I) has an overall *L* shape with the pyrazole ring being essentially coplanar with the nitro benzene ring (dihedral angle of 3.6 (2)°) and approximately perpendicular to the trifluoro phenyl ring (dihedral angle of 88.5 (2)°) and it shows no π-π interactions in the molecular packing. All bond lengths and angles are normal.

### Experimental

The method of Zhong *et al.* (2005) was used to prepare 5-amino- 3-cyano-1-[2,6-dichloro-4-(trifluoromethyl)phenyl]pyrazole(2.5 mmol). This was followed by reaction with 4-nitrobenzaldehyde(2.5 mmol) and hydrochloric acid(2 ml) in anhydrous ethanol(5 ml) to obtain 1-[2,6-dichloro-4-(trifluoromethyl)phenyl]-3-cyano- 5-[(4-nitrophenyl)methyleneamino]-1*H*-pyrazole, which was then reacted with *N*-bromosuccinimide(1.5 mmol) (Philippe, 2000) in acetonitrile (6 mL) at room temperature. After being stirred a few minutes, the reaction was monitored by TLC until all starting materials were consumed. Finally, the reaction mixture was evaporated under reduced pressure to provide the required crude product, which was then partitioned between dichloromethane and water. Seperating and drying the organic phase and evaporating it under reduced pressure gave the title compound (88.5% yield). Colourless single crystals suitable for X-ray analysis were obtained by slow evaporation of an anhydrous ethanol-acetone (2:1) solution of (I) (m.p. 463–465 K). IR (KBr, ν cm^-1^): 3396, 2244, 1623, 1596, 1525, 1384, 1310, 1135, 877, 817; ^1^H NMR (C_3_D_6_O, δ, p.p.m.): 9.60 (s, 1H), 8.34 (s, 2H), 8.16(d, 2H), 8.13 (d, 2H); ^13^C NMR (C_3_D_6_O, δ, p.p.m.): 166.4, 151.5, 147.7, 140.7, 137.4, 136.4, 134.6 (q, J = 34.1 Hz), 131.4(2 C), 130.3, 127.2 (2 C), 127.1 (2 C), 124.9(2 C), 121.4 (q, J = 271.1 Hz), 112.3.

### Refinement

All H atoms were initially located in a difference Fourier map but were eventually placed in their geometrically idealized positions and constrained to ride on their parent atoms, with C—H = 0.93 Å, and with *U*_iso_(H) = 1.2_eq_(C). The low *U*_eq_ of C1 as compared to its neighbours may be attributed to the high displacement parameters for atoms F1, F2 and F3, indicating either large thermal motion or rotational disorder of the trifluoromethyl group. However, attempts to represent the CF3 group using a disordered model were unsuccessful.

### Crystal data

**Table:**

C_18_H_7_BrCl_2_F_3_N_5_O_2_	*F*_000_ = 1048
*M**_r_* = 533.10	*D*_x_ = 1.732 Mg m^−^^3^
Monoclinic, *P*2_1_/*n*	Melting point = 463–465 K
Hall symbol: -P 2yn	Mo *K*α radiation λ = 0.71073 Å
*a* = 8.0925 (7) Å	Cell parameters from 3580 reflections
*b* = 13.0406 (10) Å	θ = 2.6–24.2º
*c* = 19.7165 (16) Å	µ = 2.33 mm^−^^1^
β = 100.753 (2)º	*T* = 298 (2) K
*V* = 2044.2 (3) Å^3^	Blcok, colorless
*Z* = 4	0.27 × 0.24 × 0.22 mm

### Data collection

**Table:**

Bruker APEX area-detector diffractometer	4437 independent reflections
Radiation source: fine-focus sealed tube	3331 reflections with *I* > 2σ(*I*)
Monochromator: graphite	*R*_int_ = 0.024
*T* = 298(2) K	θ_max_ = 27.0º
φ and ω scans	θ_min_ = 1.9º
Absorption correction: multi-scan(SADABS; Bruker, 2002)	*h* = −7→10
*T*_min_ = 0.572, *T*_max_ = 0.629	*k* = −15→16
12155 measured reflections	*l* = −25→19

### Refinement

**Table:**

Refinement on *F*^2^	Secondary atom site location: difference Fourier map
Least-squares matrix: full	Hydrogen site location: inferred from neighbouring sites
*R*[*F*^2^ > 2σ(*F*^2^)] = 0.041	H-atom parameters constrained
*wR*(*F*^2^) = 0.110	*w* = 1/[σ^2^(*F*_o_^2^) + (0.059*P*)^2^] where *P* = (*F*_o_^2^ + 2*F*_c_^2^)/3
*S* = 1.07	(Δ/σ)_max_ = 0.004
4437 reflections	Δρ_max_ = 0.56 e Å^−^^3^
280 parameters	Δρ_min_ = −0.38 e Å^−^^3^
Primary atom site location: structure-invariant direct methods	Extinction correction: none

### Special details

**Table:**

Geometry. All e.s.d.'s (except the e.s.d. in the dihedral angle between two l.s. planes) are estimated using the full covariance matrix. The cell e.s.d.'s are taken into account individually in the estimation of e.s.d.'s in distances, angles and torsion angles; correlations between e.s.d.'s in cell parameters are only used when they are defined by crystal symmetry. An approximate (isotropic) treatment of cell e.s.d.'s is used for estimating e.s.d.'s involving l.s. planes.
Refinement. Refinement of *F*^2^ against ALL reflections. The weighted *R*-factor *wR* and goodness of fit *S* are based on *F*^2^, conventional *R*-factors *R* are based on *F*, with *F* set to zero for negative *F*^2^. The threshold expression of *F*^2^ > σ(*F*^2^) is used only for calculating *R*-factors(gt) *etc*. and is not relevant to the choice of reflections for refinement. *R*-factors based on *F*^2^ are statistically about twice as large as those based on *F*, and *R*- factors based on ALL data will be even larger.

### Fractional atomic coordinates and isotropic or equivalent isotropic displacement parameters (Å^2^)

**Table:**

	*x*	*y*	*z*	*U*_iso_*/*U*_eq_	
Br1	−0.24016 (4)	0.51690 (2)	0.761142 (16)	0.05284 (13)	
Cl1	0.30689 (11)	0.82810 (7)	0.73346 (4)	0.0691 (2)	
Cl2	−0.11238 (11)	0.88488 (7)	0.91210 (4)	0.0726 (3)	
F1	0.5843 (3)	1.0868 (2)	0.92604 (16)	0.1220 (10)	
F2	0.3925 (4)	1.19099 (18)	0.89073 (15)	0.1444 (13)	
F3	0.4114 (4)	1.12171 (18)	0.98766 (12)	0.1088 (9)	
O1	0.5960 (3)	0.4599 (3)	1.17024 (13)	0.0896 (8)	
O2	0.4901 (5)	0.3126 (3)	1.14333 (19)	0.1335 (14)	
N1	0.0002 (3)	0.78718 (16)	0.79313 (11)	0.0449 (5)	
N2	−0.1070 (3)	0.81212 (17)	0.73411 (12)	0.0509 (6)	
N3	−0.4315 (4)	0.7201 (2)	0.61178 (18)	0.0856 (10)	
N4	0.0797 (3)	0.65801 (17)	0.87633 (11)	0.0467 (5)	
N5	0.4987 (4)	0.4039 (3)	1.13390 (16)	0.0754 (8)	
C1	0.4224 (5)	1.1057 (3)	0.92265 (17)	0.0655 (9)	
C2	0.3130 (4)	1.0194 (2)	0.89024 (15)	0.0510 (7)	
C3	0.3571 (4)	0.9687 (2)	0.83475 (15)	0.0538 (7)	
H3	0.4544	0.9866	0.8189	0.065*	
C4	0.2546 (4)	0.8908 (2)	0.80309 (13)	0.0469 (6)	
C5	0.1103 (3)	0.86405 (19)	0.82729 (13)	0.0437 (6)	
C6	0.0705 (3)	0.9163 (2)	0.88322 (14)	0.0488 (6)	
C7	0.1700 (4)	0.9942 (2)	0.91509 (16)	0.0521 (7)	
H7	0.1417	1.0290	0.9525	0.062*	
C8	−0.3258 (4)	0.7239 (2)	0.65789 (17)	0.0581 (8)	
C9	−0.1943 (4)	0.7263 (2)	0.71809 (14)	0.0469 (6)	
C10	−0.1445 (3)	0.6474 (2)	0.76518 (14)	0.0436 (6)	
C11	−0.0177 (3)	0.6883 (2)	0.81450 (13)	0.0427 (6)	
C12	0.0866 (4)	0.5653 (2)	0.89369 (15)	0.0538 (7)	
H12	0.0246	0.5182	0.8638	0.065*	
C13	0.1866 (4)	0.5268 (2)	0.95842 (15)	0.0482 (7)	
C14	0.1918 (4)	0.4225 (2)	0.96947 (17)	0.0607 (8)	
H14	0.1278	0.3790	0.9375	0.073*	
C15	0.2915 (4)	0.3823 (3)	1.02788 (18)	0.0652 (9)	
H15	0.2954	0.3119	1.0357	0.078*	
C16	0.3844 (4)	0.4479 (3)	1.07395 (15)	0.0546 (7)	
C17	0.3788 (4)	0.5522 (3)	1.06511 (15)	0.0578 (7)	
H17	0.4417	0.5952	1.0977	0.069*	
C18	0.2781 (4)	0.5918 (2)	1.00707 (15)	0.0535 (7)	
H18	0.2714	0.6624	1.0005	0.064*	

### Atomic displacement parameters (Å^2^)

**Table:**

	*U*^11^	*U*^22^	*U*^33^	*U*^12^	*U*^13^	*U*^23^
Br1	0.0565 (2)	0.04456 (18)	0.0567 (2)	−0.00804 (13)	0.00875 (13)	−0.00358 (13)
Cl1	0.0809 (6)	0.0743 (6)	0.0569 (5)	0.0042 (5)	0.0252 (4)	−0.0109 (4)
Cl2	0.0723 (5)	0.0821 (6)	0.0717 (5)	−0.0225 (4)	0.0347 (4)	−0.0172 (4)
F1	0.0760 (16)	0.126 (2)	0.158 (3)	−0.0348 (16)	0.0070 (15)	−0.0421 (19)
F2	0.199 (3)	0.0628 (14)	0.136 (2)	−0.0535 (17)	−0.061 (2)	0.0372 (15)
F3	0.145 (2)	0.1075 (18)	0.0751 (15)	−0.0630 (17)	0.0242 (14)	−0.0331 (13)
O1	0.0723 (17)	0.137 (3)	0.0546 (15)	0.0176 (17)	0.0000 (13)	0.0003 (17)
O2	0.166 (3)	0.101 (2)	0.113 (3)	0.027 (2)	−0.026 (2)	0.045 (2)
N1	0.0517 (13)	0.0395 (12)	0.0419 (12)	−0.0013 (10)	0.0048 (10)	0.0010 (9)
N2	0.0591 (15)	0.0435 (13)	0.0465 (13)	0.0051 (11)	0.0004 (11)	−0.0022 (10)
N3	0.081 (2)	0.080 (2)	0.082 (2)	0.0017 (17)	−0.0216 (18)	0.0013 (17)
N4	0.0533 (13)	0.0445 (13)	0.0422 (12)	−0.0015 (11)	0.0084 (10)	0.0041 (10)
N5	0.077 (2)	0.093 (2)	0.0571 (18)	0.0245 (19)	0.0151 (15)	0.0174 (18)
C1	0.073 (2)	0.062 (2)	0.059 (2)	−0.0211 (17)	0.0039 (16)	0.0027 (16)
C2	0.0598 (19)	0.0473 (15)	0.0438 (16)	−0.0091 (13)	0.0048 (13)	0.0050 (13)
C3	0.0537 (18)	0.0585 (18)	0.0501 (17)	−0.0082 (14)	0.0125 (13)	0.0068 (14)
C4	0.0545 (16)	0.0482 (16)	0.0383 (14)	0.0027 (13)	0.0094 (12)	0.0007 (12)
C5	0.0527 (16)	0.0368 (14)	0.0400 (14)	−0.0006 (12)	0.0050 (11)	0.0003 (11)
C6	0.0515 (16)	0.0502 (16)	0.0454 (15)	−0.0058 (13)	0.0110 (12)	−0.0012 (12)
C7	0.0639 (19)	0.0473 (16)	0.0452 (17)	−0.0051 (13)	0.0107 (14)	−0.0064 (12)
C8	0.0629 (19)	0.0487 (17)	0.0580 (19)	0.0000 (14)	−0.0008 (16)	−0.0009 (14)
C9	0.0501 (16)	0.0427 (15)	0.0466 (16)	0.0030 (13)	0.0056 (12)	−0.0041 (12)
C10	0.0467 (15)	0.0389 (14)	0.0454 (15)	−0.0012 (12)	0.0092 (11)	−0.0040 (11)
C11	0.0499 (15)	0.0367 (13)	0.0437 (15)	0.0006 (12)	0.0142 (12)	0.0011 (11)
C12	0.0571 (18)	0.0459 (16)	0.0546 (18)	−0.0024 (14)	0.0008 (13)	0.0003 (13)
C13	0.0485 (16)	0.0471 (16)	0.0487 (16)	−0.0013 (13)	0.0085 (12)	0.0050 (13)
C14	0.0630 (19)	0.0482 (17)	0.066 (2)	−0.0078 (15)	−0.0009 (15)	0.0060 (14)
C15	0.073 (2)	0.0504 (18)	0.073 (2)	0.0031 (16)	0.0138 (17)	0.0167 (16)
C16	0.0543 (17)	0.0666 (19)	0.0442 (16)	0.0074 (15)	0.0126 (13)	0.0091 (14)
C17	0.0634 (19)	0.0645 (19)	0.0441 (16)	0.0011 (16)	0.0067 (13)	−0.0040 (14)
C18	0.0657 (19)	0.0458 (16)	0.0490 (17)	0.0014 (14)	0.0106 (14)	−0.0009 (13)

### Geometric parameters (Å, °)

**Table:**

Br1—C10	1.865 (3)	C3—H3	0.9300
Cl1—C4	1.717 (3)	C4—C5	1.386 (4)
Cl2—C6	1.731 (3)	C5—C6	1.384 (4)
F1—C1	1.322 (4)	C6—C7	1.374 (4)
F2—C1	1.279 (4)	C7—H7	0.9300
F3—C1	1.318 (4)	C8—C9	1.439 (4)
O1—N5	1.206 (4)	C9—C10	1.394 (4)
O2—N5	1.209 (4)	C10—C11	1.382 (4)
N1—N2	1.355 (3)	C12—C13	1.466 (4)
N1—C11	1.373 (3)	C12—H12	0.9300
N1—C5	1.424 (3)	C13—C14	1.378 (4)
N2—C9	1.330 (3)	C13—C18	1.386 (4)
N3—C8	1.127 (4)	C14—C15	1.380 (4)
N4—C12	1.256 (3)	C14—H14	0.9300
N4—C11	1.380 (3)	C15—C16	1.366 (5)
N5—C16	1.474 (4)	C15—H15	0.9300
C1—C2	1.499 (4)	C16—C17	1.371 (5)
C2—C7	1.377 (5)	C17—C18	1.375 (4)
C2—C3	1.381 (4)	C17—H17	0.9300
C3—C4	1.384 (4)	C18—H18	0.9300
			
N2—N1—C11	113.7 (2)	N3—C8—C9	177.9 (4)
N2—N1—C5	118.7 (2)	N2—C9—C10	112.8 (2)
C11—N1—C5	127.5 (2)	N2—C9—C8	119.5 (2)
C9—N2—N1	103.2 (2)	C10—C9—C8	127.6 (3)
C12—N4—C11	120.3 (2)	C11—C10—C9	105.6 (2)
O1—N5—O2	123.7 (3)	C11—C10—Br1	129.0 (2)
O1—N5—C16	118.9 (3)	C9—C10—Br1	125.4 (2)
O2—N5—C16	117.4 (4)	N1—C11—N4	117.6 (2)
F2—C1—F3	107.5 (3)	N1—C11—C10	104.7 (2)
F2—C1—F1	106.3 (3)	N4—C11—C10	137.6 (2)
F3—C1—F1	103.1 (3)	N4—C12—C13	123.8 (3)
F2—C1—C2	113.6 (3)	N4—C12—H12	118.1
F3—C1—C2	113.1 (3)	C13—C12—H12	118.1
F1—C1—C2	112.5 (3)	C14—C13—C18	119.8 (3)
C7—C2—C3	121.7 (3)	C14—C13—C12	118.1 (3)
C7—C2—C1	119.7 (3)	C18—C13—C12	122.1 (3)
C3—C2—C1	118.5 (3)	C13—C14—C15	120.2 (3)
C2—C3—C4	119.1 (3)	C13—C14—H14	119.9
C2—C3—H3	120.4	C15—C14—H14	119.9
C4—C3—H3	120.4	C16—C15—C14	118.7 (3)
C3—C4—C5	120.1 (3)	C16—C15—H15	120.6
C3—C4—Cl1	119.6 (2)	C14—C15—H15	120.6
C5—C4—Cl1	120.3 (2)	C15—C16—C17	122.4 (3)
C6—C5—C4	119.1 (2)	C15—C16—N5	118.3 (3)
C6—C5—N1	120.4 (2)	C17—C16—N5	119.2 (3)
C4—C5—N1	120.5 (2)	C16—C17—C18	118.6 (3)
C7—C6—C5	121.6 (3)	C16—C17—H17	120.7
C7—C6—Cl2	119.3 (2)	C18—C17—H17	120.7
C5—C6—Cl2	119.1 (2)	C17—C18—C13	120.2 (3)
C6—C7—C2	118.3 (3)	C17—C18—H18	119.9
C6—C7—H7	120.9	C13—C18—H18	119.9
C2—C7—H7	120.9		
